# Host genes and infectious diseases.

**DOI:** 10.3201/eid0403.980322

**Published:** 1998

**Authors:** J McNicholl

**Affiliations:** Centers for Disease Control and Prevention, Atlanta, Georgia, USA.


### Host Genes and Infectious Diseases

**Janet McNicholl**
Centers for Disease Control and Prevention, Atlanta, Georgia, USA

This panel presented data on host genes that influence susceptibility to or manifestations of four infectious diseases: Puumala hantavirus infection, tuberculosis (TB), Lyme disease, and AIDS. Gus Birkhead, Council of State and Territorial Epidemiologists, introduced the session, highlighting its timeliness in relation to the rapidly emerging body of data on our 100,000 human genes that stems from the Human Genome and related projects.

The presentations introduced several approaches to identifying a host gene–infectious disease interaction. Panelists presented case-control studies of hantavirus infection, AIDS, TB, and Lyme disease that used the candidate gene approach; the new approach, genome scanning by microsatellites to identify genes associated with TB susceptibility, was also described. Candidate genes were chosen on the basis of pathology of the infectious disease (human leukocyte antigen [HLA], tumor necrosis factor [TNF], the antigen processing [TAP]), mouse genetic studies of the pathogen (*NRAMP1* in TB), or epidemiologic findings of disease severity (Vitamin D and TB).

### Susceptibility-Associated Major Histocompatibility Complex (MHC) Haplotype in Severe Puumala Hantavirus Infection

Annti Vaheri, Haartman Institute, University of Helsinki, described the epidemiology of hantavirus infections in Northern Europe. The pathogens, enveloped RNA viruses primarily of the Puumala and Dobrava genotypes, are carried by rodents such as mice and voles and cause a range of disease in humans. While the epidemics in the United States are of hantaviruses that cause primarily pulmonary disease, in northern Europe, renal disease is the primary pathologic manifestation, as evidenced by increased capillary permeability, infiltrates of CD8+ T cells, high levels of ICAM-1, and expression of TNF-α and transforming growth factor (TGF)-ß. Although most infections with these viruses are probably subclinical or cause mild disease, in 10% of patients disease may progress to shock, 5% may require dialysis, and some may die. Of those who recover, renal damage may later result in chronic hypertension. Because hantaviruses are variable and are usually transmitted as swarms of viruses, it was proposed that host factors, such as HLA genes, might influence the spectrum of disease. Indeed, this has been shown to be the case. Persons who express the HLA-B8 genes had more severe disease with lower blood pressures, higher creatinine (1), and more virus in the urine and blood by polymerase chain reaction (PCR) (2). Persons with HLA-B27 had milder disease (3). The finding of TNF-α expression in the kidney of infected patients prompted an analysis of the TNF 1 and 2 alleles (at positions -308 and -238) by restriction fragment length polymorphism (RFLP), and as might have been predicted from their linkage to the HLA-A1-B8-DR3 haplotype, nearly all who progressed to shock expressed the TNF 2 allele (M. Kanerva, unpub. data). This allele has been linked to high TNF production (4).

Because the HLA-A1-B8-DR3 MHC haplotype is associated with insulin-dependent diabetes mellitus and other autoimmune diseases that may have a viral etiology, it was asked if molecular mimicry could explain the association of this haplotype with the renal disease of Puumala virus infection. Dr. Vaheri stated that no such evidence exists and that the association probably reflects a propensity to a particular type of immune response that results in disease. Whether the genetic associations observed with Puumala hantavirus disease are due to a primary association with the TNF 2 allele or the linked HLA alleles is not known and deserves future research. Another important field is the mapping of HLA-restricted epitopes in hantaviruses.

### Host Susceptibility to TB in Africa

Although TB has been present in human populations for millennia, its reemergence as a public health problem and the new tools of molecular genetics have provided an impetus to study host genetic susceptibility to TB disease.

Richard Bellamy, Wellcome Trust Center for Human Genetics, presented studies that used both candidate- and genome-screening approaches to define these factors in African populations. As stated during the question-and-answer session, many studies of TB should be considered studies of TB disease rather than TB susceptibility, since most persons, particularly in Africa, are TB infected, but (at least in HIV-negative populations) fewer than 10% become ill. Dr. Bellamy's studies were carried out in populations with low HIV prevalence, HIV-infected persons were excluded, and disease was defined as smear-positive TB. Historically, in most populations, particularly in The Gambia and South Africa, the sources of patients and controls for these studies, TB is predominantly a disease of males. Previous studies of mono- and dizygotic twins have also suggested a genetic component (reviewed in 5).

One of the studies described by Dr. Bellamy used new tools from the human genome and other projects called microsatellite markers (intronic sections of cytosine, adenine repeats) and automated robotic DNA typing using four-color fluorescent labels with 20 markers per lane of large gels that are scanned and analyzed by software such as GeneScan 672 and Genotyper 1.2. He analyzed 92 sibling-pairs from The Gambia and South Africa. Cosegregation with TB was identified for markers on chromosomes 3, 5, 6, 8, 9, 15, and the X chromosome. A second study of 83 sibling-pairs from the same countries again linked the same sites on Xq and 15p with lod scores of >2. While these studies do not identify the genes in question, further studies of these regions may reveal the relevant genes (e.g., the microsatellite region identified on Xq is close to genes encoding the CD40 ligand and human LAMP).

Bellamy's group identified two additional genes associated with TB in candidate gene association studies of African TB cases and ethnically matched controls (6). The human homologue *NRAMP1* of the mouse *Bcg* gene that confers resistance to bacillus Calmette-Guérin has been located on chromosome 2q35. Four polymorphisms in *NRAMP1* were studied with microsatellite markers and probes that distinguished single-base substitutions and a 4-bp deletion in the gene. While all four polymorphisms were associated with TB, two, one intronic and another in the 3' untranslated region, were particularly overrepresented in TB patients; persons heterozygous for INT4 GC and 3'UTR deletion had a fourfold increased risk of having TB (6). The 3'UTR allele is of unusually high prevalence in the West African population studies but is uncommon in Europeans. This may partly explain the higher susceptibility to TB in African Americans compared with other ethnic groups. While the physiologic function of *NRAMP1* has not been defined, it may affect phagolysosome function. Dr. Bellamy's data suggest that the polymorphisms they have defined or linked polymorphisms may alter *NRAMP1* function and therefore the host's ability to clear intracellular pathogens. In vitro studies to address the effect of these polymorphisms on macrophage function are in progress.

Dr. Bellamy also presented unpublished data on vitamin D receptor genotypes and susceptibility to TB disease. This gene was chosen because of clinical and laboratory data suggesting vitamin D may be important in host defenses against TB (7,8). He observed a low prevalence of the homozygous t vitamin D receptor genotype in TB cases but not in controls. This genotype is also associated with increased risk for osteoporosis (9,10). These findings raise the question whether administering vitamin D to populations at risk for TB disease might be a simple public health measure to reduce the disease. However, the effect of such therapy might be hard to estimate because of the low prevalence of the tt homozygous genotype.

The genes (e.g., HLA) identified in these and other studies are certainly not the only genes involved in host susceptibility to TB. Dr. Bellamy estimated that together they account for less than 2% of the total familial clustering effect in this disease.

### HLA and the Pathogenesis of Lyme Arthritis

Host responses to another bacterium, the spirochete *Borrelia burgdorferi*, and the clinical spectrum of Lyme arthritis were discussed by Allen Steere, Department of Rheumatology and Immunology, New England Medical Center, Boston, Massachusetts. Another vector-borne human pathogen, *B. burgdorferi* causes a multisystem disease that may affect the skin, nervous system, heart, or joints. Arthritis is a major late manifestation of the illness. Although all manifestations are usually treatable with antibiotic therapy, approximately 10% of patients with Lyme arthritis have persistent joint inflammation for months or even years after antibiotic therapy. In these patients, PCR tests for *B. burgdorferi* DNA in joint fluid have been negative after antibiotic treatment, which suggests that joint inflammation may sometimes continue after the spirochete has been eradicated from the joint.

Dr. Steere's group is studying host factors that may be important in the pathogenesis of chronic, treatment-resistant Lyme arthritis. Studies of HLA class II alleles have shown that HLA-DRB1*0401 alleles are associated with chronic Lyme arthritis and lack of response to antibiotic therapy (11). This allele is also associated with an increased risk of developing severe rheumatoid arthritis (12). In a study of antibody responses in patients throughout the course of Lyme disease, immunoglobulin G (IgG) responses to outer-surface protein A (OspA) and OspB of the spirochete often developed near the beginning of prolonged episodes of arthritis (13). Arthritis lasted considerably longer after treatment in patients with HLA-DR4 and OspA and OspB antibody reactivity than in those who lacked responses to these proteins (13). The cellular arm of the immune response has also been examined by Dr. Steere's group, and persons with treatment-resistant Lyme arthritis usually have T cells that react with many OspA epitopes, whereas treatment-responsive patients usually do not. A possible explanation for these findings is that the T-cell response to OspA in patients with treatment-resistant Lyme arthritis may cross-react with a self antigen in the joint, and the response to this self antigen may continue to cause joint inflammation for months or even years after the eradication of the spirochete from the joint.

How does one treat patients with Lyme arthritis who do not appear to respond to therapy? Dr. Steere recommended that if they have not responded to antibiotics after 2 months and the PCR test on joint fluid is negative for *B. burgdorferi* DNA, patients should be treated with antiinflammatory agents. When asked whether HLA genes might influence Osp-based vaccines for Lyme disease, Dr. Steere noted that studies to address this question have not yet been carried out.

### Host Genes, HIV Susceptibility, and Disease Course

The rapidly growing and complex body of knowledge on the host genes that influence susceptibility to HIV infection and progression to AIDS was reviewed by Richard Kaslow, Department of Epidemiology, University of Alabama, Birmingham, Alabama. The studies reported by Kaslow and others in the last 2 years have greatly benefited from several longitudinal cohort studies, some focusing on HIV-infected seroconverters or HIV-exposed persons in the United States and Europe. More than 10 years after these cohorts have been established, adequate power to address the role of candidate genes in transmitting HIV horizontally and vertically and in affecting the rate of disease progression has been obtained, while increased knowledge of HIV's mode of cellular entry has provided new candidate genes to study. HIV enters cells through an interaction with both CD4 and a chemokine receptor of the 7 Tm family (14). Dr. Kaslow first reviewed the role of genes in encoding chemokine receptors (CCR5 and CCR2) and chemokines (SDF-1) in HIV disease. While CCR5 has multiple allelic variants in its coding region (15), the deletion of a 32-bp segment results in a nonfunctional receptor (reviewed in 16), thus preventing HIV entry; two copies of this gene provide strong protection against HIV infection in epidemiologic studies, although the protection is not absolute. This gene is found in up to 20% of Europeans but is rare in Africans and Asians. Multiple studies of HIV-infected persons have shown that presence of one copy of this gene delays progression to AIDS by about 2 years. A mutation in another chemokine receptor gene, that coding for CCR2, has also been reported by several groups to be associated with a delayed progression to AIDS (reviewed in 17). This polymorphism (a position 64 Val→Ile substitution) does not appear likely to affect receptor function, and the mutation may be linked to another polymorphism in the promotor of CCR5 (18). Nevertheless, studies of persons with both CCR2 64I polymorphism and CCR5 delta 32 deletion suggest the effect of both genes on HIV disease progression is additive (19). A polymorphism in the chemokine SDF-1, which binds to another HIV entry receptor, CXCR4, also delays HIV progression and similarly appears additive to the effects of the CCR2 and CCR5 polymorphisms (20).

Dr. Kaslow also reviewed studies of the HLA system (at the Class I HLA A, B, C and Class II DR and DQ and the antigen processing [TAP] loci) and how complex combinations of different HLA alleles alter the risk of developing AIDS in several cohorts of HIV-infected persons (21). The effects of different combinations of HLA alleles appear to delay HIV progression by a variable number of years and to be additive to the effects of the chemokine gene polymorphisms described above.

These new findings about HIV and host genes have led to new approaches to AIDS treatments, such as those directed at chemokine receptors, and hold great promise for advancing our ability to combat this disease.

### References

1. Mustonen J, Partanen J, Kanerva M, Pietilä K, Vapalahti O, Pasternack A, et al. Genetic susceptibility to severe course of nephropathia epidemica caused by Puumala hantavirus. Kidney Int 1996;49:217-21.
2. Plyusnin A, Hörling J, Kanerva M, Mustonen J, Cheng Y, Partanen J, et al. Puumala hantavirus genome in patients with nephropathia epidemica: correlation of PCR positivity with HLA haplotype and link to viral sequences in local rodents. J Clin Microbiol 1997;35:1090-6.
3. Mustonen J, Partanen J, Kanerva M, Pietilä K, Vapalahti O, Pasternack A, et al. Asssociation of HLA B27 with benign clinical course of nephropathia epidemica caused by Puumala hantavirus. Scand J Immunol. In press 1998.
4. Wilson AG, Symons JA, McDowell TL, McDevit HO, Duff GW. Effects of polymorphism in the tumor necrosis factor alpha promoter on transcriptional activation. Proc Natl Acad Sci U S A 1997;94:3195-9.
5. Bloom BR, Small PM. Editorial. The evolving relation between humans and *Mycobacterium tuberculosis*. N Engl J Med 1998;338:677-8.
6. Bellamy R, Ruwende C, Tumani C, McAdam PWJ, Whittle HC, Hill AVS. Variations in the *NRAMP1* gene and susceptibility to tuberculosis in West Africans. N Engl J Med 1998;338:640-4.
7. Davies PD. A possible link between vitamin D deficiency and impaired host defence to *Mycobacterium tuberculosis*. Tubercle 1985;66:301-6.
8. Rook GA, Steele J, Fraher L, Barker S, Karmali R, O'Riordan J, et al. Vitamin D3, gamma interferon, and control of proliferation of *Mycobacterium tuberculosis* by human monocytes. Immunology. 1986;57:159-63.
9. Sainz J, Van Tornout JM, Loro ML, Sayre J, Roe TF, Gilsanz V. Vitamin D-receptor gene polymorphisms and bone density in prepubertal American girls of Mexican descent. N Engl J Med. 1997;337(2):77-82.
10. Morrison NA, Qi JC, Tokita A, Kelly PJ, Crofts L, Nguyen TV, et al. Prediction of bone density from vitamin D receptor alleles. Nature 1994;367(6460):284-287.
11. Steere AC, Dwyer E, Winchester R. Association of chronic Lyme arthritis with HLA-DR4 and HLA-DR2 alleles. N Engl J Med 1990;323:219-23.
12. Gregerson PK, Silber J, Winchester RJ. The shared epitope hypothesis: an approach to understanding the molecular genetics of rheumatoid arthritis. Arthritis Rheum 1987;30:1205-13.
13. Kalish RA, Leong JM, Steere AC. Association of treatment-resistant chronic Lyme arthritis with HLA-DR4 and antibody reactivity with OspA and OspB of *Borrelia burgdorferi*. Infect Immun 1993;61:2774-9.
14. Murphy PM. Chemokine receptors: structure, function and role in microbial pathogenesis. Cytokine & Growth Factor Reviews 1996;7:47-64.
15. Carrington M, Kissner T, Gerrard B, Ivanov S, O'Brien SJ, Dean M. Novel alleles of the chemokine-receptor gene CCR5. Am J Hum Genet 1997;61:1261-7.
16. McNicholl JM, Smith DK, Qari SH, Hodge T. Host genes and HIV: the role of the chemokine receptor gene CCR5 and its allele (Δ32 CCR5). Emerg Infect Dis 1997:3:261-71.
17. Garred P. Chemokine-receptor polymorphisms: clarity or confusion for HIV prognosis [editorial]. Lancet 1998;351:2-3.
18. Kostrikis LG, Huang Y, Moore JP, Wolinsky SM, Zhang L, Guo Y, et al. A chemokine receptor CCR2 allele delays HIV progression and is associated with a CCR5 promotor mutation. Nature Med 1998;4;350-3.
19. Smith MW, Dean M, Carrington M, Winkler C, Huttley GA, Lomb DA, et al. Contrasting genetic influence of CCR 2 and CCR5 variants on HIV infection and disease progression. Science 1997;277;959-65.
20. Winkler C, Modi W, Smith MW, Nelson GW, Wu X, Carrington M, et al. Genetic restriction of AIDS pathogenesis by an SDF-1 chemokine gene variant. Science 1998;279;380-93.
21. Kaslow RA, Carrington M, Apple R, Park L, Munoz A, Saah AJ, et al. Influence of combinations of human major histocompatibility complex genes on the course of HIV-1 infection. Nature Medicine 1996;2:405-11.
